# Effects of Temperature on the Meiotic Recombination Landscape of the Yeast *Saccharomyces cerevisiae*

**DOI:** 10.1128/mBio.02099-17

**Published:** 2017-12-19

**Authors:** Ke Zhang, Xue-Chang Wu, Dao-Qiong Zheng, Thomas D. Petes

**Affiliations:** a College of Life Science, Zhejiang University, Hangzhou, Zhejiang Province, China; b Ocean College, Zhejiang University, Zhoushan, Zhejiang Province, China; c Department of Molecular Genetics and Microbiology, Duke University School of Medicine, Durham, North Carolina, USA; Tel Aviv University

**Keywords:** hot spot, meiotic recombination, microarray, temperature, yeast

## Abstract

Although meiosis in warm-blooded organisms takes place in a narrow temperature range, meiosis in many organisms occurs over a wide variety of temperatures. We analyzed the properties of meiosis in the yeast *Saccharomyces cerevisiae* in cells sporulated at 14°C, 30°C, or 37°C. Using comparative-genomic-hybridization microarrays, we examined the distribution of Spo11-generated meiosis-specific double-stranded DNA breaks throughout the genome. Although there were between 300 and 400 regions of the genome with high levels of recombination (hot spots) observed at each temperature, only about 20% of these hot spots were found to have occurred independently of the temperature. In *S. cerevisiae*, regions near the telomeres and centromeres tend to have low levels of meiotic recombination. This tendency was observed in cells sporulated at 14°C and 30°C, but not at 37°C. Thus, the temperature of sporulation in yeast affects some global property of chromosome structure relevant to meiotic recombination. Using single-nucleotide polymorphism (SNP)-specific whole-genome microarrays, we also examined crossovers and their associated gene conversion events as well as gene conversion events that were unassociated with crossovers in all four spores of tetrads obtained by sporulation of diploids at 14°C, 30°C, or 37°C. Although tetrads from cells sporulated at 30°C had slightly (20%) more crossovers than those derived from cells sporulated at the other two temperatures, spore viability was good at all three temperatures. Thus, despite temperature-induced variation in the genetic maps, yeast cells produce viable haploid products at a wide variety of sporulation temperatures.

## IMPORTANCE

In the yeast *Saccharomyces cerevisiae*, recombination is usually studied in cells that undergo meiosis at 25°C or 30°C. In a genome-wide analysis, we showed that the locations of genomic regions with high and low levels of meiotic recombination (hot spots and cold spots, respectively) differed dramatically in cells sporulated at 14°C, 30°C, and 37°C. Thus, in yeast, and likely in other non-warm-blooded organisms, genetic maps are strongly affected by the environment.

## INTRODUCTION

Recombination between homologous chromosomes not only ensures proper segregation of recombined homologues to opposite poles during the first cellular division but also is an important driver of genome evolution (1). Meiotic recombination is initiated by programmed formation of double-strand DNA breaks (DSBs) that are catalyzed by Spo11p via a covalent protein-DNA intermediate (2). The genomic distribution of recombination events and DSBs in most organisms is not random, with some regions of the genome having high levels of recombination (hot spots) and others having low levels (cold spots) (3–9). The first meiotic recombination hot spot characterized in detail was the *ade6-M26* allele of *Schizosaccharomyces pombe* (10–12). In the current study, we examine the distribution of hot spots and cold spots in *Saccharomyces cerevisiae* strains that undergo meiosis at different temperatures.

There are several methods for determining the locations of hot spots and cold spots. The “classic” method is to measure the rate of meiotic crossovers (COs) as a function of the physical distance between two markers. Before the advent of whole-genome sequencing, this method of mapping hot spots and cold spots had low resolution because of the lack of markers and limited information about the physical distance between markers. In fungi, hot spots could also be identified by detecting markers that had a high level of non-Mendelian segregation by tetrad analysis. Such events, in which heterozygous markers segregate in a pattern of 1:3 or 3:1 instead of in the expected pattern of 2:2, are called “gene conversion events.” Genetic experiments demonstrated that most crossovers were associated with gene conversion near the site of the crossover (13), and subsequent genetic and physical studies demonstrated that conversion reflected the formation of a heteroduplex near the site of the recombination-initiating double-strand break, followed by the repair of mismatches within the heteroduplex (14). Thus, a high frequency of gene conversion indicated a meiotic recombination hot spot.

Both of the methods described above were unsuited for high-resolution mapping of hot spots and cold spots throughout the genome. Genome-wide methods of mapping meiosis-specific DSBs were first developed in *Saccharomyces cerevisiae*. Most of these experiments (3, 15, 16) relied on the observation that the Spo11p was covalently attached to the broken DNA ends and that this attachment persisted in strains with certain mutations (*rad50S* and *sae2*). In experiments using epitope-tagged Spo11p, the DNA at the sites of DSBs could be immunoprecipitated and hybridized to microarrays containing genomic sequences. Two groups mapped DSBs by generating probes corresponding to single-stranded regions associated with DSBs in *dmc1* mutant strains (9, 17). Pan et al. mapped meiosis-specific DSBs to single-base resolution by sequencing oligonucleotides that were covalently attached to Spo11p (8). Last, using microarrays that could detect the segregation of individual single-nucleotide polymorphisms (SNPs), Mancera et al. mapped crossovers and conversion events in spores derived from tetrads (18). In general, the different mapping methods yielded similar patterns of hot spots and cold spots with the exception that the telomeres and centromeres were colder for recombination in the *rad50S* and *sae2* strains than in the wild-type or *dmc1* strains (9, 17).

There is a general consensus about some features of *S. cerevisiae* recombination hot spots. First, there is no single sequence motif that hot spots share. Second, DSBs are more common in intergenic regions and are particularly common in intergenic regions with diverging transcripts (3); as discussed below, this relationship is likely explained by the double dose of recombination-stimulating transcription factors (TFs) in these regions. Third, DSBs associated with hot spots usually occur in regions of open chromatin (19), although the degree of openness does not correlate well with the strength of the hot spot. Fourth, the activity of some hot spots requires the binding of transcription factors (20). For example, the Bas1p transcription factor is required for the *HIS4* recombination hot spot. However, the activities of other hot spots that have a Bas1 binding site are unaffected by the absence of the protein and, for some hot spots, the activity is elevated in *bas1* strains (16, 21). Fifth, in most studies, hot spots are associated with both local and regional domains of elevated G+C content (3, 22); this correlation is weak over short (<1-kb) “windows” but is strong over longer (>3-kb) windows (8). Sixth, the level of DSB formation is proportional to the level of recombination as measured genetically (for example, by measuring gene conversion rates [23]). Seventh, high levels of meiotic recombination are often associated with elevated levels of trimethylated H3K4 (24). At high resolution, regions of H3K4me3 do not correlate very well with the location of the DSBs (25), and it has been suggested that this modification is involved in recruiting nearby chromosome regions to the chromosome axis where the Spo11 complex resides (26). In summary, the strength of hot spots is a complex function of the multiple factors described above, and our ability to predict the strength of hot spots on the basis of these factors is still limited.

The cold regions of the yeast genome appear to be regulated by large domains of chromosome structure rather than by specific local elements. Regions within 10 to 20 kb of the telomeres have low levels of DSBs, as do centromeric regions (3, 15). The rRNA gene array and regions flanking the array have low levels of meiotic exchange (27, 28). At least part of this suppression appears to be related to histone deacetylation, since *sir2* mutants, which have elevated levels of histone acetylation, have higher levels of recombination in the rRNA genes (29), as well as higher levels of DSBs in the rRNA genes and near the telomeres (28).

For most of the experiments described above, the strains were sporulated at temperatures between 22°C and 30°C; in this report, temperatures are always given in centigrade. In yeast, no genome-wide study has been performed at more-extreme temperatures, although some individual hot spots have been shown to be temperature dependent. For example, Fan et al. (23) found that the level of gene conversion at the *HIS4* hot spot was 50% when cells were sporulated at 18°C but was only 19% when cells were sporulated at 25°C. Performing experiments in a different genetic background, Cotton et al. (30) showed that the conversion rate at *HIS4* was 23% at 23°C and was about 2% at 37°C. Johnston and Mortimer (31) examined crossovers for 15 genetic intervals at 15°C and 25°C. In 13 of the 15 intervals, no significant alteration was observed, and in 2 intervals, the crossover frequency was reduced by about half at the lower temperature. In a genome-wide meiotic analysis performed with *S. pombe*, Hyppa et al. (32) used chromatin immunoprecipitation of Rec12-associated DNA (the equivalent of Spo11-associated DNA) and microarrays to examine recombination in strains sporulated at 25°C and 30°C. Ninety-six percent of the 288 hot spots were unaltered by the two temperatures. Eleven hot spots had more DSBs at 25°C than at 34°C.

A recent review summarizes studies of the effects of temperature on recombination in a wide variety of organisms (33). In general, this information is limited to single studies in each organism based on examining a small number of genetic intervals. Some studies suggest that temperature can affect recombination over large chromosomal regions. For example, in *Drosophila*, examining flies raised at temperatures ranging between 19°C and 30°C, Mather (34) found that the frequency of crossing-over was unaffected for most euchromatic chromosome regions but was elevated at the higher temperature in intervals containing heterochromatin. A similar effect was noted in barley (33).

Below, we examine the temperature-dependent patterns of meiotic recombination by two different methods: mapping of Spo11-induced DSBs using microarrays and mapping of crossovers/gene conversion events in tetrads. Our results demonstrate the existence of temperature-dependent and temperature-independent meiotic recombination hot spots. We also show that the number of crossovers is elevated in cells sporulated at 30°C compared to cells sporulated at 14°C or 37°C. This work emphasizes the important role of the environment (temperature) in genome evolution through influencing the meiotic recombination process.

## RESULTS

### Mapping meiosis-specific DSBs across the yeast genome in a diploid sporulated at 14°C, 30°C, and 37°C.

The KZ5 diploid strain was constructed by crossing two sequence-diverged haploid strains, KZ3 (W303-1A background) and KZ4 (YJM789 background). The resulting diploid, similarly to those we have used in previous studies (35), is heterozygous for about 55,000 SNPs. KZ5 is homozygous for the *SPO11-ZZ* allele, encoding an epitope-tagged version of Spo11. The ZZ epitope consists of IgG binding domains from protein A of *Staphylococcus aureus*, and proteins with this epitope can be precipitated using IgG beads (16); based on tetrad analysis, the epitope tag did not affect the frequency of meiotic recombination at the *HIS4* hot spot (16). In addition, KZ5 is homozygous for *sae2*. In strains with this mutation, following induction of meiosis-specific DSBs by Spo11p and its associated proteins, Spo11 remains covalently attached to the broken ends (36). Details of the construction of KZ5 are given in Materials and Methods.

To determine the effects of the temperature of sporulation on patterns of meiosis-specific DSBs, we sporulated the diploid KZ5 in liquid sporulation medium for 1 to 2 days at 14°C, 30°C, or 37°C (details are given in Materials and Methods). At temperatures lower than 14°C or higher than 37°C, the diploid JSC22-1 (isogenic with KZ5 except for the *sae2* and *SPO11-ZZ* alterations) produced very few tetrads, whereas 30°C is close to the optimal temperature for sporulation in a nearly isogenic derivative (18). We immunoprecipitated Spo11-associated DNA by using IgG beads as described for previous studies (16). The DNA samples were labeled with Cy5-dUTP (details are given in Materials and Methods and in reference 16) and mixed with genomic DNA isolated from vegetative cells of KZ5 that was labeled with Cy3-dUTP. This mixture was hybridized to an Agilent comparative genome hybridization (aCGH) array that included about 15,000 oligonucleotides (average size of 60 bases) distributed evenly throughout the 13-Mb genome. Thus, the markers were roughly 1 kb apart.

Using a GenePix 4000B scanner and GenePix Pro 6.0 software, we obtained the ratio of the levels of hybridization of the experimental and control samples. The relative strengths of DSBs at each genomic position were calculated as the normalized ratio of hybridization (log_2_ of S/C [with S representing Spo11-enriched DNA and C representing control DNA]); the normalization was calculated by setting the ratio of S/C such that the sum of the hybridization values for all oligonucleotides for the Spo11-enriched samples and all control DNA samples was 1 (log_2_ of 0). For each temperature at each genomic position, we obtained four measurements. These measurements were from two independent sporulated samples, with two independent immunoprecipitations from each sporulation. For each genomic site for each experiment, we determined the normalized hybridization ratio (see Data Set S1 in the supplemental material). Most (>90%) of these values were between −2 and +2. Genomic sites with these ratios represent both intergenic and intragenic regions.

10.1128/mBio.02099-17.7 DATA SET S1  Rankings of hot spots and cold spots. Download DATA SET S1, XLS file, 3.4 MB. Copyright © 2017 Zhang et al. 2017 Zhang et al. This content is distributed under the terms of the Creative Commons Attribution 4.0 International license.

We ranked hybridization ratios in two different ways, both of which are shown in Data Set S1. First, the ratios were ranked between 0 and 1, with the highest value representing the highest degree of Spo11 enrichment (details are given in Materials and Methods). Second, we ranked the ratios from 1 to 14,872 (the number of oligonucleotides on the microarray), with “1” representing the strongest hot spot.

We classified hot and cold genomic sites (oligonucleotides on the microarray) as those for which the hybridization ratio ranked in the top 10% and bottom 10%, respectively, in all four experiments (Data Set S1). This criterion should result in false assignment of a hot or cold site with a probability of <0.0001. The numbers of hot sites at 14°C, 30°C, and 37°C were 531, 509, and 437, respectively. Comparing the number of hot sites to the number of not-hot sites (not-hot sites were calculated by subtracting the number of hot sites from 14,872, the total number of oligonucleotides on the microarray) between two different temperatures by a contingency chi-square test, the resulting *P* values are 0.507 (comparison of 14°C and 30°C), 0.002 (14°C and 37°C), and 0.019 (30°C and 37°C). Thus, there were significantly fewer hot sites at 37°C than at the other two temperatures.

The criterion of choosing hot sites based on the top 10% of ranked hybridization values is similar to that used previously in other microarray studies (top 12.5%) (3, 15, 16); those other studies yielded numbers of hot sites similar to those observed in the present study, ranging between 303 and 605 for strains sporulated between 25°C and 30°C. Our use of ranked hybridization values controlled for different efficiencies of sporulation. The requirement that the hot sites must rank in the top 10% in all four experiments is conservative, minimizing the likelihood of falsely assigning a site as hot. However, our analysis emphasizes the hottest hot spots. In experiments based on sequencing Spo11p-associated oligonucleotides, Pan et al. (18) detected about 5- to 10-fold more hot regions than were observed by microarray analysis.

The numbers of cold sites at 14°C, 30°C, and 37°C were 232, 187, and 147, respectively. By the same type of calculation used for the hot sites, we determined *P* values of 0.030 (comparison of 14°C and 30°C), <0.0001 (14°C and 37°C), and 0.032 (30°C and 37°C). Elevated temperatures, therefore, decrease the number of cold sites. Classifying oligonucleotides at adjacent genomic positions as hot or cold, we integrated those positions into single hot spots or cold spots (Data Set S2). The numbers of hot spots at 14°C, 30°C, and 37°C were 312, 379, and 326, respectively, and the numbers of cold spots at 14°C, 30°C, and 37°C were 195, 176, and 139, respectively.

10.1128/mBio.02099-17.8 DATA SET S2  Locations of hot spots and cold spots at three temperatures. Download DATA SET S2, XLSX file, 0.1 MB. Copyright © 2017 Zhang et al. 2017 Zhang et al. This content is distributed under the terms of the Creative Commons Attribution 4.0 International license.

### Distributions of hot spots/cold spots in strains sporulated at different temperatures.

**FIG 1:**
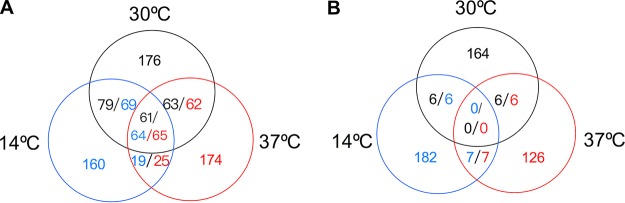
Numbers of hot spots (A) and cold spots (B) that are in common in cells sporulated at 14°C, 30°C, or 37°C. In the Venn diagrams, the numbers of hot spots/cold spots shown in blue, black, or red indicate hot spots/cold spots detected at 14°C, 30°C, or 37°C, respectively. Hot spots had hybridization rankings in the top 10th in 4 of 4 microarrays, and cold spots ranked in the bottom 10th in 4 of 4 arrays.

The locations and activities of these hot spots are listed in Data Set S2.1. Although there were similar numbers of hot spots at the three temperatures, only about 20% were hot at all three temperatures (Fig. 1A). We can classify the hot spots into seven groups: those that were hot at only one temperature (indicated as 14°C, 30°C, or 37°C hot spots), those that were hot at two temperatures (indicated as 14°C/30°C, 14°C/37°C, or 30°C/37°C hot spots), and those that were hot at all three temperatures (14°C/30°C/37°C hot spots). As expected, the classes of hot spots obtained from temperatures of sporulation that were closer together (14°C/30°C and 30°C/37°C hot spots) were about 3-fold more common than the class obtained from the more extreme temperature sporulation difference (14°C/37°C). Despite differences in the hot spots at the three temperatures, the recombination activities of the 14,872 positions represented on the microarray were very significantly correlated between any two of the temperatures (Pearson’s coefficient *r* between 0.42 and 0.52; *P* < 0.0001).

To be sure that our conclusion that only a small fraction of hot spots were hot at all three temperatures was not dependent on using the criterion of the top 10% of rankings, we also performed the same calculation while requiring the hot spots to rank in the top 5% or top 20% of rankings for all four experiments. Using rankings of the top 5%, we found 161, 180, and 160 hot spots at temperatures of 14°C, 30°C, and 37°C, respectively; only 19% of hot spots were hot at all three temperatures. Using rankings of the top 20%, we found 661, 731, and 625 hot spots at temperatures of 14°C, 30°C, and 37°C, respectively; again, only 19% of hot spots were hot at all three temperatures. Thus, our conclusions are not strongly dependent on the cutoff value chosen to be considered a hot spot.

**FIG 2:**
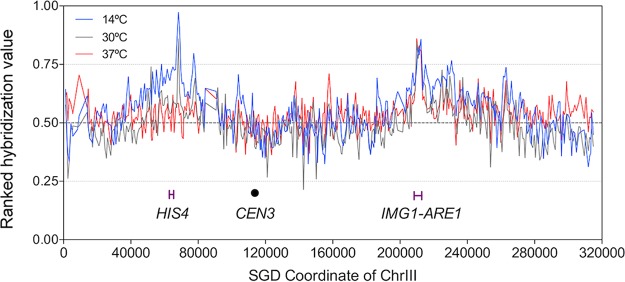
Recombination activities along chromosome III in cells sporulated at 14°C, 30°C, or 37°C. The *y* axis shows the hybridization values (log_2_ of the ratio of Spo11-enriched sample divided by control DNA sample) for each oligonucleotide on the microarray ranked from 0 (lowest) to 1 (highest). The *x* axis shows the Saccharomyces Genome Database (SGD) coordinates on chromosome III in kilobases. Blue, black, and red lines indicate the recombination activities in cells sporulated at 14°C, 30°C, and 37°C, respectively. The *HIS4* hot spot on the left arm has its highest activity at 14°C, intermediate activity at 30°C, and very little activity at 37°C. In contrast, the activity of the *IMG1-ARE1* hot spot on the right arm is unaffected by temperature.

In Fig. 2, we show the ranked hybridization values of oligonucleotides as a function of the position on chromosome III. The two most prominent hot spots are of different classes. The hot spot marked *HIS4* is of the 14°C/30°C class (HS43 in the 14°C data and HS45 in the 30°C data; Data Set S2.1), with its strongest activity in cells sporulated at 14°C. In contrast, the hot spot marked *IMG1-ARE1* is of the 14°C/30°C/37°C class (HS48 at 14°C, HS50 at 30°C, HS46 at 37°C; Data Set S2.1), with approximately the same activity in cells sporulated at all three temperatures. Table S1 in the supplemental material shows the 22 hottest open reading frames (ORFs) in cells sporulated at 30°C and their rankings at 14°C and 37°C.

10.1128/mBio.02099-17.5 TABLE S1  Comparisons of DSB activities of 22 hottest ORFs at 30°C with those of the same ORFs at other temperatures of sporulation. Download TABLE S1, DOCX file, 0.1 MB. Copyright © 2017 Zhang et al. 2017 Zhang et al. This content is distributed under the terms of the Creative Commons Attribution 4.0 International license.

One trivial explanation of the observation that many of the hot spots are temperature specific is that our measurements of hot spot activity have sufficiently large distributions that some regions that are “hot” do not pass our significance threshold. By this explanation, we expect to find that the same regions may have similar average rankings for hot spot but that, at some temperatures, the rankings have too broad a distribution to pass the significance criterion (all four measurements must rank in the top 10%) for one or more of the temperatures.

Although there are undoubtedly some hot spots in this category, many are not. Data Set S2.1 lists the hot spots identified at the three temperatures. The hot spot labeled HS39 (on chromosome II) in the 30°C data is also a hot spot at 37°C but not at 14°C (Data Set S1). The oligonucleotide within this 30°C/37°C hot spot starts at coordinate 798832. Of the 14,872 oligonucleotides for which we determined rankings, the average rankings of this oligonucleotide were 419 and 280 for the temperatures of 30°C and 37°C, respectively (based on data in Data Set S1). The average ranking for the 14°C samples was 9,687. We also did statistical comparisons of the rankings (using the values between 0 and 1) for all of the oligonucleotides (Data Set S1), comparing recombination activities between 14°C and 30°C, 30°C and 37°C, and 14°C and 37°C. For these comparisons, those with significantly (*P* < 0.05) elevated or reduced activities are designated “Up” or “Down,” respectively, in columns AN, AO, and AP of Data Set S1 (*P* values that rounded off to <0.001 are highlighted in yellow). For HS39, the hot spot activity was significantly less at 14°C than at either 30°C (*P* = 0.004) or 37°C (*P* = 0.004) by *t* test. Of the statistical comparisons done for the 30°C hot spots that were in the categories of 30°C, 14°C/30°C, and 30°C/37°C (total of 318 hot spots), *P* values of <0.01 were found for 41 comparisons and *P* values of <0.05 were found for 115 comparisons. In addition, as described below, we confirmed some of the observed differences by Southern analysis or by tetrad analysis.

### Distributions of hot spots and cold spots relative to those of the telomeres and centromeres.

**FIG 3:**
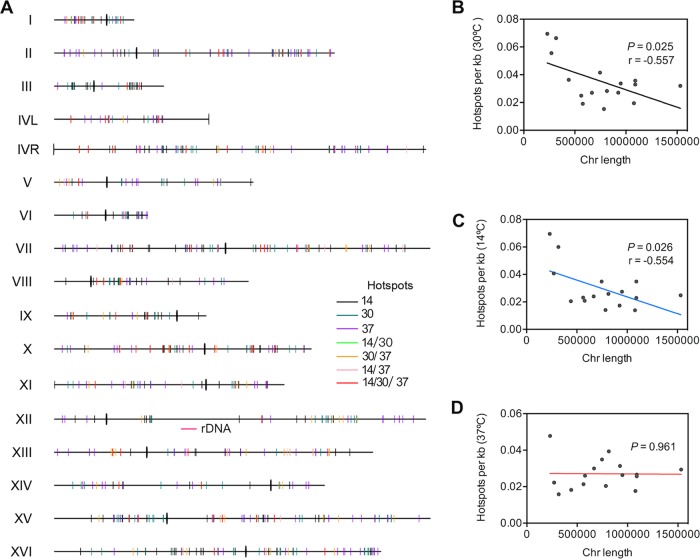
Chromosomal distribution of hot spots. (A) Distribution of various classes of hot spots on each of the 16 chromosomes. We show hot spots that have activities at only one temperature (14°C, 30°C, and 37°C), those that are active at two temperatures (14°C/30°C, 30°C/37°C, 14°C/37°C), and those that are active at all three temperatures (14°C/30°C/37°C). Centromeres are shown as thick vertical lines intersecting each chromosome. (B to D) Densities of hot spots as a function of chromosome (Chr) size in cells sporulated at 30°C (B), 14°C (C), and 37°C (D). At 30°C and 14°C, there is a significant negative correlation between chromosome size and hot spot density, although this relationship is not observed at 37°C.

The chromosomal locations of the various classes of hot spots are shown in Fig. 3A. As described in the introduction, regions near the telomeres and centromeres tend to have fewer DSBs than other regions of the genome, particularly *in rad50S* and *sae2* strains, and this pattern generally persists in our data. At 30°C, if the 379 hot spots were randomly distributed across the genome, the number of hot spots expected within 50 kb of the telomeres and within 25 kb of the centromeres would be 66 (corrected for the number of probes within these regions). Our observed number of hot spots (i.e., 387) is significantly lower than the expected value (chi-square test, *P* < 0.01). In addition, very few hot spots were observed adjacent to ribosomal DNA (rDNA) (shown as a red bar under chromosome XII in Fig. 3A), as expected from previous studies (8, 9, 16). The hot spots at 14°C and 37°C were also underrepresented within 50 kb of the telomeres or within 25 kb of the centromeres, although the underrepresentation was slightly greater at 14°C and slightly less at 37°C than at 30°C (see Fig. S1 in the supplemental material).

10.1128/mBio.02099-17.1 FIG S1  Distribution of hot spots and cold spots relative to telomeric and centromeric regions. We determined the expected numbers of hot spots and cold spots in these regions (the red portion of the leftmost bar in each diagram) by calculating the numbers of oligonucleotides on the microarray that were within 50 kb of the telomeres or within 25 kb of the centromeres. We then determined the proportions of hot spots (Fig. S2A) or cold spots (Fig. S2B) that were within these regions (red) or outside these regions (blue). The temperatures of sporulation are indicated below each bar. (A) Proportion of hot spots near telomeres and centromeres. (B) Proportion of cold spots near telomeres and centromeres. Download FIG S1, TIF file, 0.2 MB. Copyright © 2017 Zhang et al. 2017 Zhang et al. This content is distributed under the terms of the Creative Commons Attribution 4.0 International license.

10.1128/mBio.02099-17.2 FIG S2  Chromosomal distribution of cold spots. This figure is similar to Fig. 3, which shows the chromosomal distribution of hot spots. (A) Distribution of cold spots of all classes on each of the 16 yeast chromosomes. The centromeres are shown as short thick vertical lines that intersect the thin line representing the chromosome. (B to D) Density of cold spots as a function of chromosome length of cells sporulated at 30°C (Fig. S3B), 14°C (Fig. S3C), and 37°C (Fig. S3D). No significant correlations, positive or negative, were observed at any of the temperatures. Download FIG S2, TIF file, 0.4 MB. Copyright © 2017 Zhang et al. 2017 Zhang et al. This content is distributed under the terms of the Creative Commons Attribution 4.0 International license.

10.1128/mBio.02099-17.3 FIG S3  Comparison of observed and expected numbers of “hot” microarray elements (oligonucleotides) in intragenic regions and various classes of intergenic regions. The data for these graphs were obtained from Table S2. Each bar in this graph represents a ratio (the observed number of elements divided by the expected number of elements) calculated for five different classes of chromosomal sequence: total intergenic sequences; class 1 intergenic sequences, located between divergently transcribed genes; class 2 intergenic sequences, located between genes transcribed in the same direction; class 3 intergenic sequences, located between genes transcribed convergently; total intragenic sequences. Data for cells sporulated at 14°C, 30°C, and 37°C are shown by blue, black, and red bars, respectively. As noted previously, hot spots tend to be located intergenically, with a preference for class 1 intergenic regions. Download FIG S3, TIF file, 0.2 MB. Copyright © 2017 Zhang et al. 2017 Zhang et al. This content is distributed under the terms of the Creative Commons Attribution 4.0 International license.

**FIG 4:**
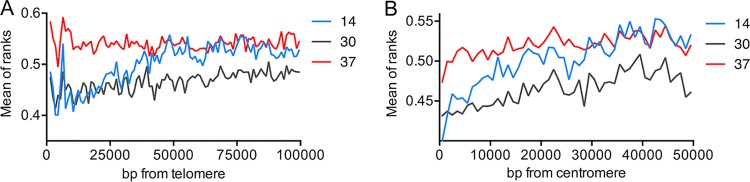
Recombination activities as a function of the distance from the telomere and centromere in cells sporulated at 14°C (blue lines), 30°C (black lines), or 37°C (red lines). (A) Recombination activity as a function of the distance from the telomere. The *y* axis shows the recombination activities (average of rankings of oligonucleotides) in a moving window of 5 kb moved 1 kb at a time. (B) Recombination activity as a function of the distance from the centromere. The analysis was performed by a procedure similar to that described for panel A.

To further determine the effect of temperature on DSBs near centromeres and telomeres, we calculated the average ranks within 100 kb of each telomere and 50 kb of each centromere for 16 chromosomes. We show these rankings for the telomeres (Fig. 4A) and centromeres (Fig. 4B) in a moving window of 1 kb. At the telomeres, suppression was clear for cells sporulated at 14°C and 30°C, but suppression was substantially weaker for cells sporulated at 37°C; this effect is also evident in Fig. 2. The suppression of DSB formation at the centromere was also less for cells sporulated at 37°C than for cells sporulated at the other two temperatures.

In contrast, and as observed in previous studies (see, for example, reference 3), cold spots were overrepresented near the telomeres and centromeres (Data Set S2 and Fig. S1 and S2). This overrepresentation was strongest at 14°C and diminished to statistical insignificance at 37°C. We observed no overlap of cold spots among cells sporulated at different temperatures (Fig. 1B). Two factors were likely responsible for this observation. First, as discussed above, the regional specificity of cold spots was substantially diminished in cells sporulated at 37°C. Second, by definition, cold spots are regions with low levels of DSB formation and, therefore, low levels of immunoprecipitated DNA. Thus, the signal-to-noise ratio in our estimates of recombination activity is likely to be higher for hot spots than for cold spots. In general, regions that were very cold when sporulated at one temperature were at least moderately cold when sporulated at other temperatures. For example, the oligonucleotide at position 607248 on chromosome II was designated a cold spot at 30°C since it had a ranking below 13,304 (bottom 10% of rankings) for all four experiments; the mean ranking was 14,186. However, the mean rankings of the same oligonucleotide in cells sporulated at the other two temperatures were also low: 12,758 and 11,864 for cells sporulated at 14°C and 37°C, respectively (Data Set S1). Nonetheless, this oligonucleotide passed the “cold spot” criterion at 30°C, but not at the other two temperatures.

### Density of hot spots and cold spots as a function of chromosome size.

Kaback et al. (37) noted that small yeast chromosomes had a higher density of crossovers than large yeast chromosomes, and Gerton et al. (3) noted a higher density of DSBs on the shorter chromosomes. Pan et al. (8) found a higher density of Spo11-associated oligonucleotides on smaller yeast chromosomes, although the density of hot spots was not significantly associated with chromosome size. In the current study, the density of hot spots in cells sporulated at 14°C and 30°C was inversely related to chromosome size, although this effect was not observed in cells sporulated at 37°C (Fig. 3B to D). In contrast, the density of cold spots was independent of chromosome size (Fig. S2).

### Association of hot and cold sites with intergenic and intragenic regions.

Based initially on Southern analysis of individual hot spots (7, 57) and subsequently on high-resolution mapping of Spo11-associated oligonucleotides throughout the genome (8), it was shown that most meiosis-specific DSBs in yeast occur between genes rather than within genes. In addition, hot spots are located between divergently transcribed genes in preference to being located between genes transcribed in the same direction or between convergently transcribed genes (3, 8). Since our analysis involves Spo11-associated fragments that are about 1 to 2 kb in size and since the density of oligonucleotides on the microarrays is about one oligonucleotide per kilobase, our study did not allow high-resolution mapping of DSBs. In general, the presence of a strong DSB site located between two genes results in an elevated signal for the oligonucleotide representing the intergenic region as well as in elevated signals for the oligonucleotides in the flanking genes. For the analysis described below, we examined the recombination activity of each oligonucleotide individually, and each oligonucleotide was classified as intragenic or intergenic.

The ratio of intergenic probes to intragenic probes on the microarray is about 1:2.7. Oligonucleotides that were scored as “hot” were significantly enriched for intergenic regions in cells sporulated at all three temperatures, with the strongest enrichment seen for the 37°C data (Table S2 and Fig. S3). The approximate ratios (intergenic to intragenic) at 14°C, 30°C, and 37°C were 1:2.1, 1:1.3, and 1:0.97, respectively. By chi-square analysis, the data corresponding to the elevated numbers of intergenic hot sites were very significant (*P* < 0.0001) for cells sporulated at 30°C and 37°C and were of borderline significance (*P* = 0.01) for cells sporulated at 14°C (Table S2); because of corrections for multiple comparisons, only *P* values of <0.003 are significant. It should be pointed out that the data reported for the number of intragenic hot sites in Table S2 likely represent overestimates, since (as discussed above) DSBs occurring in intergenic regions often result in an elevated level of recombination of flanking genes.

10.1128/mBio.02099-17.6 TABLE S2  Association of hot spots and intergenic/intragenic regions. Download TABLE S2, DOCX file, 0.1 MB. Copyright © 2017 Zhang et al. 2017 Zhang et al. This content is distributed under the terms of the Creative Commons Attribution 4.0 International license.

The ratio of oligonucleotides located in the three classes of intergenic regions (class 1, divergently transcribed; class 2, convergently transcribed; class 3, transcribed in the same direction) is 2.4:1:3.4. There was a significant elevation in the number of class 1 events and a significant reduction in the number of class 2 events compared to the expected numbers for cells sporulated at any of the three temperatures (Table S2 and Fig. S3). The relative numbers seen with classes 1, 2, and 3 are those that would be expected if hot spot activity were stimulated by the binding of transcription factors. Cold sites were overrepresented (*P* < 0.003) for class 2 events at 30°C and 37°C, but not at 14°C.

### Correlations with our previous microarray studies of hot spots.

Most of our previous studies (3, 16, 28) were performed with similar types of microarrays in cells sporulated at 25°C. The diploids used in these previous studies had few polymorphisms, and both parental strains were closely related to the commonly used S288c strain in contrast to the W303-1A/YJM789 hybrid background used in the current study. Nonetheless, many of the recombination hot spots observed in the current study share the same location as those observed in our previous study. Of the 22 hottest ORFs in the present study, 20 were hot spots in the analysis of Mieczkowski et al. (16) (Table S1); this result indicates that the large number of polymorphisms in KZ5 likely has little effect on the distribution of hot spots and cold spots. This similarity is not simply a reflection of the similar methods used to examine hot spots in these two studies. Of the 25 regions with the largest representation of Spo11p-associated oligonucleotides in the study by Pan et al. of an SK1-derived diploid (8), 20 matched the hot spots determined in our analysis for KZ5 cells sporulated at 30°C. Considering the substantial sequence differences between the diploids and the differences in the methods used in these two studies, this level of agreement is surprising.

In our previous studies, we noticed a striking positive correlation between local peaks of GC content (based on a 5-kb sliding window that moved at intervals of 1 kb) and meiotic hot spot activity assayed in cells sporulated at 25°C (3, 16); this correlation is observed in the current data at all three temperatures (*P* < 0.0001). In addition, negative correlations were observed between hot spots and nucleosome occupancy, as well as between hot spots and binding sites of Rec8p, representing meiosis-specific cohesion (8, 16). The negative correlation between nucleosome occupancy and hot spot activity was strong at 30°C (*P* < 0.001; *r* = −0.052) but was statistically insignificant in cells sporulated at 14°C (*P* = 0.064; *r* = −0.027) or 37°C (*P* = 0.174; *r* = −0.0198). In contrast, the negative correlation between hot spots and Rec8p binding was strong at all three temperatures of sporulation (*P* < 0.0001); it should be noted, however, that nucleosome occupancy and Rec8p binding were measured in cells grown at 30°C.

### Association of modified DSB activities with transcription factors (TFs).

The recombination activity of the *HIS4* hot spot requires the binding of three transcription factors (Bas1p, Bas2p, and Rap1p), but the activity is unaffected by a promoter deletion that substantially reduces the level of expression (7). One interpretation of that result is that the binding of these transcription factors created a chromatin environment that is favorable for the Spo11 machinery. A global analysis of recombination hot spots in wild-type and *bas1* strains, coupled with an examination of Bas1p binding sites, showed that, at different genomic locations, Bas1p stimulated recombination, repressed recombination, or had no effect on recombination (16, 21). Thus, the recombination-stimulating effects of TFs are dependent on chromosome context.

To determine whether any of the observed temperature-dependent alterations in hot spot activity could reflect the binding of specific TFs, we examined hot spots that had altered activities at different temperatures to determine whether they were enriched for binding sites for specific TFs. For this analysis, we restricted our analysis to ORFs that had temperature-dependent alterations in activity at 30°C compared to the other two temperatures. For these comparisons, we required that the “hot” ORF of the pair also have a “hot” promoter region. The analysis was done using the “Rank by TF” function in the Yeastract Database (http://www.yeastract.com/).

There were 34 ORFs at 14°C and 65 ORFs at 37°C that had different DSB activities in their promoter regions compared to the same regions at 30°C. When we pooled these changes without regard to which temperature resulted in the higher level of recombination activity, we found that the ORFs that were different between 14°C and 30°C were enriched (*P* < 0.05 after correction for multiple comparisons) for 21 TFs (Fig. S4). The equivalent analysis for ORFs that had different activities at 30°C and 37°C showed that 39 TFs were enriched (Fig. S4). Twelve of the TFs (Met28p, Phd1p, Met32p, Yap6p, Sok2p, Acd2p, Skn7p, Mcm1p, Msn2p, Sfp1p, Rap1p, and Rox1p) were in common (Fig. S4). It is noteworthy that three of the TFs indicated in Fig. S4 (Bas1p, Rap1p, and Gcn4) bind to the region upstream of the *HIS4*, one of the hot spots that responds most dramatically to the temperature of sporulation.

10.1128/mBio.02099-17.4 FIG S4  Transcription factors that were significantly associated with ORFs that had recombination activities at 30°C that were different from those seen at 14°C or 37°C. As described in the text, we identified ORFs that had significantly different recombination activities at 14°C than at 30°C (34 ORFs) or at 37°C than at 30°C (65 ORFs). Using the “Rank by TF” function in the Yeastract Database, we determined whether transcription factors associated with these ORFs were significantly overrepresented (*P* < 0.01, after correction for multiple comparisons). We show the expected (gray bars) and observed (red bars) ratios of targeted ORFs for these transcription factors as bar graphs. (A) Transcription factors overrepresented in ORFs that had different recombination activities at 14°C and 30°C. (B) Transcription factors overrepresented in ORFs that had different recombination activities at 30°C and 37°C. Download FIG S4, TIF file, 0.6 MB. Copyright © 2017 Zhang et al. 2017 Zhang et al. This content is distributed under the terms of the Creative Commons Attribution 4.0 International license.

### Confirmation of chromatin immunoprecipitation with microarray technology (ChIP-chip) results by Southern analysis.

**FIG 5:**
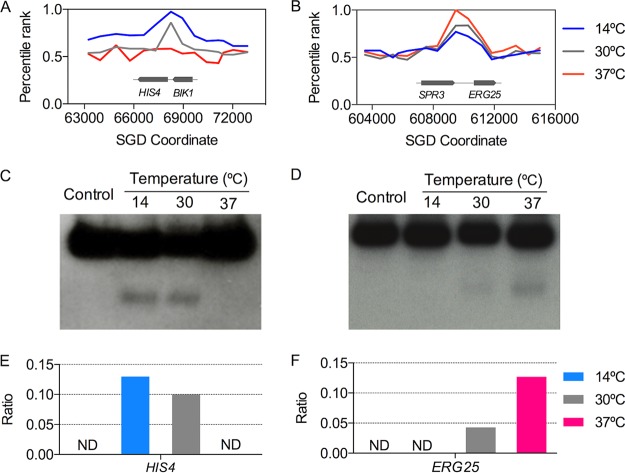
Microarray and Southern blot analysis of two recombination hot spots. (A) Recombination activities of oligonucleotides located near the *HIS4* and *BIK1* genes as determined by microarray analysis. The activity of this hot spot is strongest in cells sporulated at 14°C (blue line). (B) Recombination activities of oligonucleotides (microarray analysis) located near the *SPR3* and *ERG25* genes. The activity of this hot spot is strongest in cells sporulated at 37°C (red line). (C) *HIS4* hot spot. (D) *ERG25* hot spot. Southern blot analysis of genomic DNA isolated from control DNA (nonsporulated) and of cells sporulated at 14°C, 30°C, or 37°C was performed. Genomic DNA was digested with BglII (C) or BamHI (D). Following gel electrophoresis and DNA transfer to nylon membranes, the samples were hybridized to PCR-generated probes (details are given in Materials and Methods). The slow-migrating band in each lane is the expected size for genomic fragments without a meiosis-specific DSB, and the fast-migrating band is the expected size for the hot spot-associated DSB. (E) *HIS4*. (F) *ERG25*. We show the ratios of the meiosis-specific DNA fragment relative to the intact DNA fragment as determined with ImageJ software.

To confirm our microarray data, we examined the temperature-dependent formation of DSBs at the *HIS4* and *ERG25* hot spots by Southern analysis. Based on our microarray analysis (Data Set S1), recombination activity at the *HIS4* hot spot decreased with increasing temperatures of sporulation (Fig. 5A), whereas the *ERG25* hot spot had the opposite response (Fig. 5B). DSB frequencies, as determined by Southern analysis, were consistent with the expectations based on the microarrays (Fig. 5C to E).

### Analysis of the temperature-dependent *HIS4* hot spot by tetrad analysis.

Another method of monitoring hot spot activity is by tetrad dissection of strains heterozygous for an auxotrophic marker located near the hot spot. In a diploid heterozygous marker in gene *A* (alleles *A* and *a*) that is not located near a hot spot, most (>95%) tetrads segregate 2 *A* spores to 2 *a* spores. Gene conversion events in which sequence information is transferred nonreciprocally between homologues result in tetrads with 3*A*:1*a* or 1*A*:3*a* patterns of segregation (14). Most meiotic conversion events involve repair of a DSB through a pathway of heteroduplex formation followed by repair of mismatches in the heteroduplex. In a wild-type strain, most mismatches are efficiently repaired. However, small “hairpin” loops in heteroduplexes are inefficiently repaired, resulting in postmeiotic segregation (PMS) events (38). In strains heterozygous for an auxotrophic mutation, such events can be detected by replica plating of the colonies derived from tetrad-dissection-to-omission medium. The most common PMS patterns are tetrads with 2 *A* spore colonies, 1 *a* spore colony, and 1 sectored *A/a* spore colony (5*A*:3*a* segregation) or with 1 *A* spore colony, 2 *a* spore colonies, and 1 sectored *A/a* spore colony (3*A*:5*a* segregation).

We used tetrad analysis to examine the frequency of aberrant segregation at the *HIS4* locus in cells sporulated at different temperatures. The strain used in this experiment (KZ8; construction described in Materials and Methods) is isogenic to KZ5 (the strain used for the microarray studies) except for alterations introduced by transformation. The most relevant alterations are that the strain does not have the *SPO11-ZZ* genes, is homozygous for wild-type *HIS3* alleles, and is heterozygous for the *his4-lopc* mutation. The *his4-lopc* mutation is a 26-bp palindromic sequence that results in a poorly repaired mismatch if present in a heteroduplex (38). We sporulated KZ8 at different temperatures for 3 to 7 days, and tetrads were dissected. The numbers of tetrads of various classes of *HIS4* segregants (His^+^:His^−^) were 21 for 3:1, 9 for 1:3, 1 for 4:0, 2 for 0:4, and 313 for 2:2 at 14°C; 21 for 3:1, 14 for 1:3, 1 for 4:0, and 440 for 2:2 at 30°C; and 11 for 3:1, 3 for 1:3, and 292 for 2:2 at 37°C. The numbers of aberrant segregants at 14°C and 30°C were significantly greater than the numbers observed at 37°C (*P* values of 0.016 and 0.02, respectively, by Fisher exact tests). The proportions of aberrant segregants at 14°C, 30°C, and 37°C were 9.5%, 7.3%, and 4.6%, respectively. These results are consistent with our conclusions about the temperature dependence of the *HIS4* hot spot based on microarray and Southern analyses.

Previously, we examined the aberrant segregation rates of *his4-lopc* in a strain with a different genetic background sporulated at either 18°C or 25°C (23). There were two substantive differences from the results of our current study. First, most (70% to 80%) of the aberrant segregants represented PMS events rather than conversions. Second, the rates of aberrant segregation in the study reported by Fan et al. (23) (50% aberrant segregation at 18°C and 19% at 25°C) were much higher than in the present study. The first discrepancy has a simple explanation. If a heteroduplex has an efficiently repaired mismatch near an inefficiently repaired mismatch, the excision tract extending from the efficiently repaired mismatch frequently includes the inefficiently repaired mismatch, reducing or eliminating PMS (39). Located within the 100-bp sequences flanking the insertion site of *his4-lopc* (+467), there are five SNPs that distinguish the *HIS4* genes on the two homologues, one of which is only 5 bp from *his4-lopc*. It is less clear why KZ8 has a lower frequency of *HIS4* aberrant segregation than the strain used in previous studies. It is possible that the sequence differences between the two homologues at the DSB site reduce the probability of a conversion event, although the good spore viability of strains with this genetic background (described below) argues against a strong general recombination-suppressing effect of the heterologies. A more likely explanation is that some other aspect of the genetic background (for example, the strain-specific recombination-stimulating activities of one of the polymorphic *HIS4* transcription factors) has a locus-specific effect on the frequency of aberrant segregation at the *HIS4* hot spot.

### Whole-genome mapping of meiotic recombination events.

Our microarray studies monitored DSB formation throughout the genome but did not directly examine crossovers and gene conversions. Previously, Mancera et al. (18) used SNP-specific microarrays to examine crossovers and gene conversions in DNA samples isolated from tetrads of a diploid that was closely related to the diploid used in our studies. We used this approach to analyze patterns of recombination in the diploid JSC22-1 sporulated at 14°C, 30°C, or 37°C. JSC22-1 is isogenic with KZ5 except that it is homozygous for the wild-type *SPO11* allele instead of the *SPO11-ZZ* allele.

For these experiments, cells of JSC22-1 were grown on solid rich-nutrient medium overnight and were then sporulated on solid medium at 14°C (7 days), 30°C (3 days), or 37°C (4 days). The proportions of cells that formed tetrads at 14°C, 30°C, and 37°C were 8.1%, 27.1%, and 14.4%, respectively. The proportion of viable spores at 37°C (1,208/1,540; 78%) was significantly (*P* < 0.0001 by chi-square test) lower than that at 30°C (2,059/2,324; 89%) or 14°C (1,464/1,656; 88%). The numbers of tetrads with zero, one, two, three, or four viable spores, respectively, were 2, 11, 45, 61, or 295 for 14°C sporulation; 3, 10, 67, 89, or 412 for 30°C sporulation; and 6, 26, 58, 114, or 181 for 37°C sporulation. The number of tetrads with three viable spores was significantly (*P* < 0.0001 by chi-square test) elevated at 37°C relative to the other two temperatures. Although we have not investigated the cause of this elevation, a high frequency of tetrads with three viable spores could reflect an increase in meiosis II nondisjunction.

Using SNP-specific microarrays, we genotyped a total of 84 spores derived from complete tetrads of JSC22-1, 5 tetrads at 14°C, 11 at 30°C, and 5 at 37°C. We utilized microarrays containing 25-base oligonucleotides that distinguished SNPs derived from the haploid parental strains (W303-1A or YJM789) used to construct JSC22-1 (35); about 13,000 SNPs were represented on the array, allowing us to map events to a resolution of about 1 kb. The details of DNA isolation, hybridization conditions, and analysis of the microarrays are given in Materials and Methods.

### Crossovers and gene conversions.

**FIG 6:**
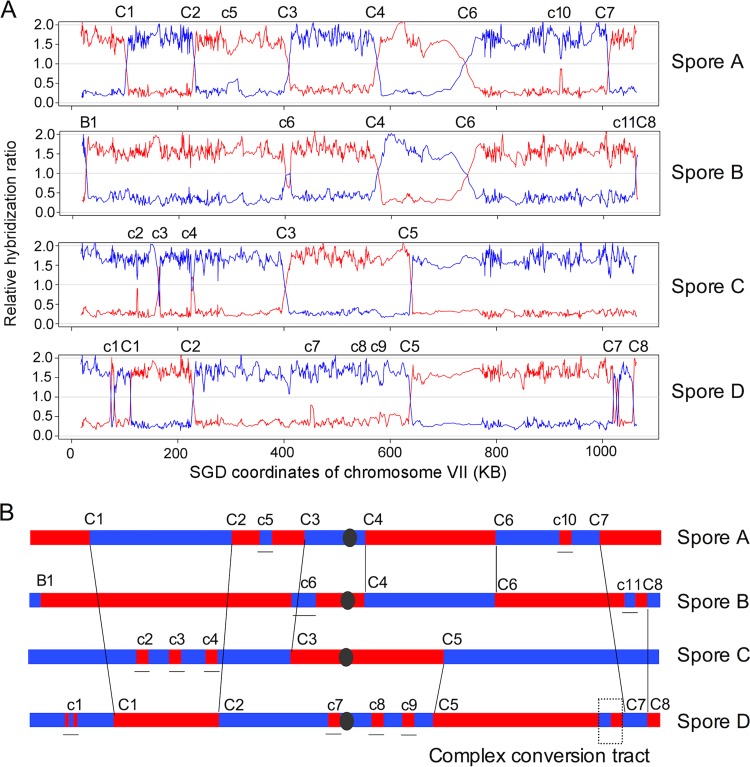
Microarray analysis of crossovers and conversions unassociated with crossovers in tetrads of JSC22. As described in the text, the diploid was constructed by crossing sequenced diverged strains W303-1A and YJM789. We dissected tetrads from the diploid sporulated at different temperatures and examined genomic DNA of the spores by using microarrays that could distinguish SNPs derived from the two genetic backgrounds. The data shown in this figure were from tetrad 30-9 (30°C sporulation, tetrad 9; Data Set S3). Only data from chromosome VII are shown. (A) SNP microarray analysis of chromosome VII. The red and blue lines show hybridization to W303-1A-specific and YJM789-specific SNPs, respectively. The *y* axis shows the hybridization ratios relative to the fully heterozygous control strain. In segments of the chromosome that contain W303-1A-derived sequences, the red line has a hybridization ratio of about 1.7 and the blue line a hybridization ratio of about 0.2. These ratios are shown in moving windows containing nine SNPs that are moved one SNP at a time. Transitions with a capital “C” followed by a number show crossovers. Transitions with a small “c” followed by a number indicate conversion events unassociated with crossovers, and the single event labeled “B” is a BIR event. It should be emphasized that conversion tracts that involve a single SNP (described in Data Set S3) are not detectable in the low-resolution images in panel A but are depicted in panel B. (B) Depictions of crossovers and conversions based on the SNP array analysis. The conversion event labeled “c1” is complex (similar to type 3 in Data Set S4), as is the conversion tract associated with C7. The numbers of the events match those shown in panel A.

A determination of the classes of meiotic recombination event requires analysis of the SNP patterns in all four spores of the tetrad. In Fig. 6A, we show the patterns of hybridization on chromosome VII in spore DNA samples from a tetrad (30-9) of a 30°C sporulation. The multiple transitions between W303-1A-derived sequences (shown in red) and YJM789-derived sequences (shown in blue) are numbered; transitions with the same number on different chromatids represent crossovers. Numbers in the schematic depiction in Fig. 6B correspond to the same events in Fig. 6A. Since the hybridization ratio is shown in a running window of nine oligonucleotides, gene conversion events that included only a small number of SNPs are not visible in Fig. 6A; these events are visible, however, when the hybridization ratios are examined at the level of individual SNPs.

10.1128/mBio.02099-17.9 DATA SET S3  Locations and types of meiotic recombination events in tetrads. Download DATA SET S3, XLSX file, 0.4 MB. Copyright © 2017 Zhang et al. 2017 Zhang et al. This content is distributed under the terms of the Creative Commons Attribution 4.0 International license.

10.1128/mBio.02099-17.10 DATA SET S4  Depictions of types and numbers of meiotic recombination events. Download DATA SET S4, XLSX file, 0.04 MB. Copyright © 2017 Zhang et al. 2017 Zhang et al. This content is distributed under the terms of the Creative Commons Attribution 4.0 International license.

**FIG 7:**
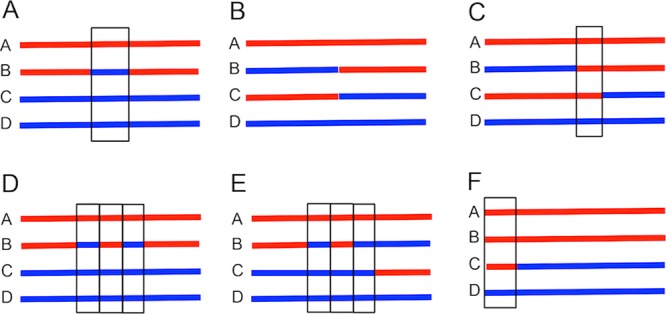
Main classes of meiotic recombination events in JSC22. In these diagrams, each line represents a specific chromosome in one spore, and the four lines are spores from a single tetrad. Red and blue lines indicate sequences derived from W303-1A and YJM789, respectively. Boxed intervals show gene conversion or BIR events. These classes match those described in Table 1. (A) Class 1 (simple conversion unassociated with crossover). In the figure, we show a conversion event in which W303-1A sequences are lost and YJM789-derived sequences are duplicated. In spore B, there are two transitions between red and blue SNPs. Events in which YJM789-derived sequences are lost and W303-1A sequences are duplicated were found with similar frequencies (Table 1). (B) Class 2 (crossover unassociated with a conversion). In events in this class, in the spores that have a crossover (spores B and C), the transition between red and blue sequences occurs between the same two SNPs. (C) Class 3 (crossover associated with a simple conversion). In events in this class, the regions flanking the conversion event in spores B and C are reciprocally recombined. There is a single transition between the red and blue SNPs in each of these spores, but the transitions are in different places. (D) Class 4 (complex conversion unassociated with a crossover). In events in this class, the conversion events are not associated with a crossover of flanking sequences and there are more than two transitions between the SNPs on the chromatid with the conversion (spore B). There are a number of different types of class 4 (Table 1 and Data Set S4). (E) Class 5 (complex conversions associated with crossover). As described for panel D, the conversion tract (spore B) has more than two transitions. In addition, the sequences flanking the conversion tract in spores B and C are reciprocally recombined. (F) Class 6 (break-induced replication [BIR]). In events in this class, there is a long (>5-kb) terminal conversion tract (spore C).

We divided individual recombination events into six classes, illustrated in Fig. 7. Class 1 events are simple gene conversions unassociated with crossovers. In this class, at a specific position, one chromatid (the B chromatid in Fig. 7A) has a small region derived from a nonsister chromatid, resulting in a 3:1 segregation pattern, considering all four chromatids. An example of a class 1 event is designated “c2” in Fig. 6. The region of gene conversion, usually less than 5 kb in size, is outlined with a rectangle in Fig. 7. For class 1 events, there are two transitions between the locations of SNPs derived from one homologue and SNPs derived from the other. The four coordinates that define these transitions are listed in Data Set S3. There are two subclasses of the class 1 events: those with three W303-1A-derived sequences and one YJM789-derived sequence and those with one W303-1A-derived sequence and three YJM789-derived sequences (see, for example, Fig. 7A). These subclasses are called “type 1” and “type 2,” respectively, in Data Set S4, which tabulates the numbers of events in all subclasses of recombination events. In many previous studies of recombination, it was shown that the chromatid with the initiating DSB acts as a recipient in the conversion event. Thus, as indicated in Fig. 7A, we infer that the initiating DSB occurred on the B chromatid. The numbers of type 1 and type 2 class 1 events in cells sporulated at the two temperatures are shown in Table 1. As shown in this table, class 1 events constituted similar fractions of the total recombination events in cells sporulated at all three temperatures, and the W303-1A- and YJM789-derived homologues were equally susceptible to meiosis-specific DSBs (comparison of the percentages of type 1 and type 2 events).

**TABLE 1:** Number of recombination events of different classes in cells sporulated at different temperatures^a^

Class	Type(s)	Class description	No. of events (% of total)		
			14°C	30°C	37°C
1	1	Simple conversion, no crossover; duplication of W303-1A-derived sequences	77 (13)	184 (12)	71 (13)
1	2	Simple conversion, no crossover; duplication of YJM789-derived sequences	92 (15)	209 (13)	67 (13)
1	1 + 2 (class 1 total)	Simple conversion, no crossover	169 (28)	393 (25)	138 (26)
2	5	Crossover, no detectable conversion	199 (33)	544 (35)	180 (34)
3	7	Simple conversion plus crossover; duplication of W303-1A-derived sequences	97 (16)	267 (17)	97 (18)
3	6	Simple conversion plus crossover; duplication of YJM789-derived sequences	83 (14)	299 (19)	92 (17)
3	6 + 7 (class 3 total)	Simple conversion plus crossover	180 (30)	566 (36)	189 (36)
4	3 + 4	Complex conversion, no crossover	3 (0.5)	3 (0.2)	0 (0)
5	8–25	Complex conversion plus crossover	42 (7)	63 (4)	21 (4)
6	26 + 27	Break-induced replication (BIR)	6 (1)	6 (0.4)	0 (0)
					
Total			599	1,575	528

^a^ The calculations of the numbers of events were based on an analysis of cells sporulated at 14°C (5 tetrads), 30°C (11 tetrads), and 37°C (5 tetrads).

Crossovers represented about 70% to 74% of the total recombination events for cells sporulated at all temperatures (sum of classes 2, 3, and 5; Table 1). Crossovers with no detectable conversion event (class 2; 33% to 35% of the total) and crossovers adjacent to simple conversions (class 3; 30% to 36% of the total) were much more common than crossovers associated with complex conversions (class 5; 4% to 7% of the total) (Fig. 7 and Table 1). The two smallest classes consisted of complex gene conversion events unassociated with crossovers (class 4; <1% of the total) and break-induced replication (BIR) events (class 6; <1% of the total). The distinction between simple and complex gene conversion events is that simple conversions have a single continuous block of homology transferred between homologues (Fig. 7C) whereas complex conversion events (Fig. 7D and E) have two or more blocks of homology transferred. We considered an event to be a complex conversion, rather than two adjacent simple conversion events, if the distance between transitions was less than 7.5 kb.

Excluding the BIR events, we observed the following numbers of conversions unassociated with crossovers (classes 1 and 4) and 95% confidence limits (smallest and largest numbers) per tetrad for each temperature as follows: for 14°C, 34 + 13 (range, 20 to 48); for 30°C, 36 + 15 (range, 19 to 103), for 37°C, 28 + 11 (range, 18 to 39). The comparable numbers of crossovers (classes 2, 3, and 5) per tetrad for each temperature were as follows: for 14°C, 84 + 11 (range, 73 to 96); for 30°C, 107 + 12 (range, 86 to 149); for 37°C, 78 + 20 (range, 62 to 101). As in the analysis by Mancera et al. (18), we assume that we detected all crossovers but that we missed those gene conversion events that were unassociated with crossovers if they did not include one or more of the oligonucleotide sequences represented on the microarray. Since the SNPs represented on our microarray are separated by an average distance of about 1 kb (35) and the median meiotic conversion tract length is about 2 kb (18), the fraction of such undetected conversions is likely to be substantial. We estimated the fraction of undetected gene conversion events using the method described by Mancera et al. (18). For each temperature, we determined the fraction of crossovers that had a detectable conversion event and divided the number of conversions unassociated with crossovers (classes 1 and 4) by that fraction. For example, at 30°C, 0.536 of the crossover events were associated with conversions. Since the number of conversions unassociated with crossovers was 396, the corrected number was 396/0.536, or 739; the corrected number of conversions unassociated with crossovers per tetrad was 67.

Using the correction factor, we estimated that the average numbers of conversions unassociated with crossovers and the average numbers of crossovers per tetrad (the sums of the two events are indicated in parentheses) were 65 and 84 (149) at 14°C, 67 and 107 (174) at 30°C, and 54 and 78 (132) at 37°C. One important point concerning these data is that the numbers of recombination events per tetrad differed by less than 35% at the different sporulation temperatures despite alterations in which genomic regions are hot spots. In addition, at all three temperatures, all chromosomes had at least one crossover (Data Set S3), i.e., the obligatory crossover required to ensure accurate chromosome disjunction at meiosis I. Our estimate of 174 recombination events per tetrad in cells sporulated at 30°C is similar to the observed total of 157 events in a similar genetic background in cells sporulated at about 24°C by Mancera et al. (18). The level of crossovers was significantly lower at 14°C and 37°C than at 30°C (*P* values less than 0.01, as determined with the Mann-Whitney test). In addition, using uncorrected numbers for conversions and crossovers, we found that the average number of recombination events was significantly lower at 37°C than at 30°C (*P* value of 0.03). These observations correlate well with our estimates of the numbers of hot spots in strains at 14°C, 30°C, and 37°C described above (312, 379, and 326, respectively).

Although most of the conversion events were simple, we also observed complex conversions (classes 4 and 5 in Table 1). In conversion events unassociated with crossovers, the ratios of complex conversions to simple conversions were low (<0.02): 3/169 (14°C), 3/393 (30°C), and 0/138 (37°C). In contrast, for the conversion events associated with crossovers, the ratios of complex conversions to simple conversions were >0.11: 42/180 (14°C), 63/566 (30°C), and 21/189 (37°C). A similar effect was detected previously in experiments done with a strain similar to the one used in our study (18), although such an effect was not observed in a different genetic background (40). In addition, we found that there were significantly more complex conversion events at 14°C than at 30°C (*P* = 0.001 by Fisher exact test) or 37°C (*P* = 0.01). Interpretations of these observations are discussed below.

We observed small numbers of terminal gene conversion events (Fig. 6 and 7F), 6 at 14°C and 6 at 30°C (Table 1), which were classified as BIR events. One such event is designated “B1” in Fig. 6. Similar events have been observed previously (18, 41). A likely interpretation of these events is that a DSB located near the end of the chromosome was repaired by a nonreciprocal event in which the centromere-containing broken end invaded the opposite homologue and duplicated sequences centromere-distal to the break (42).

### Crossover interference.

In yeast, as in most eukaryotes, a meiotic crossover event reduces the probability of occurrence of an additional crossover event nearby (22, 43); this phenomenon is termed “crossover interference” or “chiasma interference.” To assess the degree of interference that was temperature dependent, we measured interference using the tetrad-randomization method employed by Mancera et al. (18). In brief, our estimates of the number of crossover events per tetrad at each temperature were used to place crossovers randomly in the genome (details are given in Materials and Methods) using 1,000 simulations for each temperature. The median distances between adjacent crossovers from these simulations were used as the expected values for comparisons to the median values obtained for the midpoints of our observed crossovers. At all three temperatures, the observed intercrossover distances significantly (*P* < 0.0001 by Mann-Whitney test) exceeded the intercrossover distance predicted if there were no interference. The observed versus the expected distances for cells sporulated at 14°C, 30°C, and 37°C were 109 kb versus 86 kb for 14°C, 83 kb versus 68 kb for 30°C, and 104 kb versus 87 kb for 37°C. Thus, crossover interference was present in strains sporulated at all three temperatures. However, the degree of interference (the gap between the observed intercrossover and randomized intercrossover distances) at 14°C was greater than that seen at 30°C or 37°C (*P* < 0.0001 by Mann-Whitney test), suggesting that interference can be affected by temperature.

### Gene conversion lengths and the genomic effect of gene conversion.

The median sizes of conversion tracts associated with crossovers at 14°C, 30°C, and 37°C were 2,937, 3,010, and 2,871 bp, respectively; these lengths were not significantly different by the Mann-Whitney test. The median tract sizes of conversions unassociated with crossovers at 14°C, 30°C, and 37°C were also not significantly different, with median lengths of 3,598, 3,327, and 3,280 bp, respectively. In previous studies, Mancera et al. (18) found that the lengths of the conversion tracts associated and unassociated with crossovers were 2 kb and 1.8 kb, respectively. These tract lengths are shorter than those observed in our study presumably because they examined meiotic segregants using microarrays that allowed detection of more SNPs than the arrays used in our study. Nonetheless, we conclude that different temperatures of sporulation do not have a strong effect on the length of conversion tracts. Since the distance between oligonucleotides on the microarray averaged about 1 kb, alterations in average tract lengths that were less than 300 bp would be difficult to detect in our study.

Although the conversion tracts were of similar lengths in strains sporulated at different temperatures, sporulated at 14°C, the diploid had about 2-fold-higher levels of complex conversion tracts than samples sporulated at the other two temperatures (11.4% at 14°C, 6.4% at 30°C, and 6.0% at 37°C; Table 1); these differences were significant by chi-square analysis (*P* < 0.02). This difference may reflect that the enzymes involved in DNA synthesis and/or strand transfer are less processive at low temperatures, resulting in more strand switching between different templates.

Mancera et al. (18) estimated that about 2% of the genome was altered by gene conversion for each meiosis event. By summing the lengths of all gene conversion tracts for the tetrads derived from each temperature and dividing by the genome size, we determined that 1% of the genome underwent conversion at 14°C, 1.2% underwent conversion at 30°C, and 0.85% underwent conversion at 37°C. The proportions of the genome undergoing gene conversion at 14°C and 37°C were significantly reduced relative to 30°C (*P* < 0.0001 by chi-square analysis).

## DISCUSSION

Many strains of the yeast *S. cerevisiae* are capable of sporulating at a wide range of temperatures, but the effects of this variable on patterns of meiotic recombination had not been systematically examined prior to our study. Although we previously showed that a genomic region located upstream of *HIS4* had 2-fold-stronger hot spot activity at 18°C than at 25°C (23, 38), no other temperature-dependent hot spots in yeast have been characterized. Our analysis described above showed that many hot spots, as well as regions that are not hot spots, are strongly affected by the temperature of sporulation. Thus, meiotic recombination maps vary depending on the temperature of sporulation.

There were two categories of temperature-dependent changes in recombination observed in our analysis: changes that affect individual genes and global alterations in recombination near the telomeres and centromeres. These two categories of alterations are discussed separately. The first point that should be emphasized is that the temperature-dependent alterations in the activities of individual genes/hot spots were not affected in a consistent direction. Some genes (for example, *HIS4*) were hottest at 14°C, and others (for example, *ERG25*) were hottest at 37°C. This result argues that Spo11p and the other proteins involved in initiating meiosis-specific DSBs function reasonably well within the temperature range tested.

As outlined in the introduction, DSB formation of individual genomic regions is regulated at two levels. First, most DSBs occur in promoter regions of genes localized in arrays of loops, and these events are stimulated by the binding of transcription factors (as discussed in more detail below). Second, trimethylated H3K4 genomic regions become tethered to the chromosome axes where Spo11p and other proteins required for DSB formation are localized (26). The evidence that transcription factor binding was required for hot spot activity and DSB formation was originally based on the analysis of the *ade6-M26* hot spot of *S. pombe* (10–12) and the *HIS4* hot spot of *S. cerevisiae* (20, 44). For *ade6-M26*, the relevant transcription factor is Atf-Pcr1 (12), and for *HIS4*, the relevant factors are Bas1p, Bas2p, and Rap1p (20, 44). Subsequently, many other hot spots defined by short sequence motifs that require transcription factors for hot spot activity were identified in *S. pombe* (45, 46). In this regard, it should be emphasized that, although hot spots in *S. cerevisiae* and *S. pombe* are not defined by a single long sequence motif, many hot spots share short motifs that bind specific transcription factors (47). In mammals, a large subset of recombination hot spots is defined by a sequence motif that binds the histone methyltransferase PRDM9 (48).

Based on these considerations, several nonexclusive models can explain the differences in hot spot activities at different temperatures. First, different recombination-promoting transcription factors could have different optimal binding temperatures that result in temperature-dependent DSBs near their binding site. A related interpretation is that the level of recombination-stimulating transcription factors varies with the temperature. This interpretation is quite likely since many yeast genes show temperature-dependent alterations in transcript levels. For example, about 500 genes are upregulated and 500 downregulated when yeast cells are exposed to low temperatures (49). An alternative possibility is that the levels of the recombination-promoting histone modification trimethylated H3K4 differ at different loci at different temperatures of sporulation.

In the present study, as in previous studies of meiotic recombination in *rad50S* or *sae2* strains (3), DSB formation was substantially reduced in pericentric regions and in regions within 20 to 50 kb of the telomere. In wild-type strains, the degree of DSB suppression is restricted to a region of about 10 kb at the centromere (9, 17), although crossovers between homologues are restricted in a larger pericentric region. The mechanism by which recombination is suppressed in this region is unclear; however, it is dependent on the Ctf19 kinetochore protein and is partially independent of pericentric cohesion (50). Similarly, the mechanism responsible for suppression of meiotic exchange near the telomere is unclear. It is unlikely to be related to telomeric silencing of gene expression, since loss of Sir2 (required for silencing) has only a small effect on meiotic recombination near the telomere (28). It is possible that the observed suppression of meiotic recombination reflects an aspect of clustering of centromeres and telomeres (perhaps resulting in a chromatin modification) that occurs prior to DSB formation. Previously, Börner et al. found that wild-type strains sporulated at 33°C had accelerated formation of DNA recombination intermediates and a stronger leptotene recombination checkpoint than strains sporulated at 23°C (51); these differences could exert context-dependent effects on the frequency of DSB formation.

One important function of meiotic recombination is to generate genetic diversity. We found that the number of crossovers per tetrad was higher at 30°C than in tetrads derived from sporulation at the other two temperatures. The smaller number of crossovers at 14°C might be a consequence of the higher degree of crossover interference, although this factor does not explain the difference in the numbers of crossovers at 30°C and 37°C. The reduced spore viability observed at 37°C relative to 30°C may be a consequence of the reduced levels of crossovers. Alternatively, the reduced level of suppression of crossovers near the centromere in cells sporulated at 37°C could elevate nondisjunction, because crossovers near the centromere elevate the frequency of nondisjunction (2).

Despite the altered patterns of recombination described above, some general features of recombination are temperature independent. The numbers of crossovers and conversions per tetrad vary <40% at the various temperatures. In tetrads obtained from all temperatures, all chromosomes get at least one crossover, and gene conversion tracts do not show significant variations in length. Thus, many of the basic properties of meiotic exchange are conserved in the temperature range of 14°C to 37°C. For organisms that experience substantial variation in environmental temperatures, this conservation is presumably evolutionarily advantageous.

It has long been observed that the meiotic recombination maps in humans are different for males and females (52, 53). Although there are many possible mechanisms to explain these differences, we suggest the possibility that the temperature at which meiosis occurs in the different sexes may be one important factor since the temperature in the testes is lower than the temperature in the ovaries.

## MATERIALS AND METHODS

### Strains and medium.

All *S. cerevisiae* strains used in this study are isogenic with the previously described haploid strains JSC12-1 (*MAT***a** *leu2-3,112 his3-11,15 ura3-1 ade2-1 trp1-1 can1-100*::*natMX4 RAD5 IV1510386*::*kanMX6-can1-100*) and JSC21-1 (*MAT*α *ade2-1 ura3 gal2 ho*::*hisG CAN1*::*natMX4 IV1510386*::*SUP4-o*) (54), except for changes introduced by transformation. JSC22-1 is a diploid strain obtained by mating JSC12-1 and JSC21-1. We inserted an epitope tag (the ZZ epitope of protein A of *Staphyloccocus aureus*) at the C terminus of *SPO11* by PCR amplification of the *SPO11 ZZ*::*K.1.URA3* gene of strain JG106 (16) using primers SPO-ZZUS (AGCATAGCCCTAAATTATAC) and SPO-ZZUA (TCCTTACATGGCTTATAACT). Strains KZ3 and KZ4 were derivatives of KZ1 and K2, respectively, in which the *SAE2* gene was replaced by *hphMX4*. These replacements were performed using PCR fragments obtained by amplifying plasmid pAG32 (55) with primers dSAE2S and dSAE2A. Mating KZ3 and KZ4 generated the KZ5 diploid strain that was used in the ChIP-chip analysis. KZ6 was constructed by transforming JSC21-1 with a DNA fragment containing the wild-type *HIS3* gene, generated by PCR amplification of genomic DNA derived from *HIS3* strain YJM785 using primers HIS3S (TAGTCAGGGAAGTCATAACAC) and HIS3A (CACCTATCACCACAACTAACT). KZ7 is a derivative of KZ6 with the *his4-lopc* allele and was constructed by two-step transplacement with plasmid pDN13; for the transformation, pDN13 was treated with restriction enzyme SnaBI. The KZ8 diploid was obtained by crossing of KZ7 and JSC21-1.

Standard rich growth medium (yeast extract-peptone-dextrose [YPD]) was used to culture yeast cells. Presporulation medium contains 5 g/liter yeast extract, 10 g/liter peptone, 1.7 g/liter yeast nitrogen base, 10 g/liter potassium acetate, 5 g/liter ammonium sulfate, and 10 g/liter potassium hydrogen phthalate. Liquid sporulation medium contains 1 g/liter yeast extract and 10 g/liter potassium acetate. Solid sporulation medium contains 1 g/liter yeast extract, 10 g/liter potassium acetate, 0.5 g/liter glucose, and 5 mg/liter adenine.

### Detection of meiotic DSBs by ChIP-chip.

Because of the *sae2* mutation, in diploid strain KZ5, Spo11p is not removed from the DNA ends. KZ5 cells were cultured in sporulation medium at 14°C, 30°C, or 37°C. The Spo11p-associated DNA was prepared by immunoprecipitation using methods described in our previous study (16). The Spo11p-enriched DNA was then PCR amplified, and the PCR products were labeled with Cy5-dUTP. Nonenriched genomic DNA that had been amplified was labeled with Cy3-dUTP and was used as a control for the CGH microarrays (Agilent custom ChIP-on-Chip 8x15k array G4499A). We acquired the microarray images with a GenePix 4000B scanner and analyzed the images with GenePix Pro 6.0 software. Subsequent steps in the data analysis were previously described by Mieczkowski et al. (16). Raw data are available at GEO (see below). The relative strengths of DSBs for each SNP were determined using the normalized ratio of hybridization (log_2_ of S/C [with S representing Spo11-enriched DNA and C representing control DNA]). To compare the recombination activities of the 14,872 individual SNPs on the microarrays, we used two methods. First, we ranked the normalized ratios of hybridization from highest (which was given a value of 1) to lowest (which was given a value of 14,872). Second, we ranked the ratios of hybridization from 0 (the lowest ratio) to 1 (the highest ratio). Intermediate ratios were assigned a rank based on the following formula: rank = [(log_2_ S/C for sample) − (log_2_ S/C for lowest-ranked sample)]/[log_2_ S/C for highest-ranked sample) − (log_2_ S/C for lowest-ranked sample)].

### Confirmation of DSBs by Southern blotting.

To induce Spo11-catalyzed DSBs, we incubated KZ5 cells in 200 ml presporulation medium at 30°C for 16 h and then transferred the cells into 200 ml sporulation medium at 14°C for 48 h, 30°C for 24 h, or 37°C for 24 h. Genomic DNA from KZ5 cells was isolated from meiotic and premeiotic cells (from presporulation medium) as described by St. Charles et al. (35). Hybridization probes were prepared by PCR amplification of genomic DNA using a commercial digoxigenin (DIG) DNA labeling kit (Roche).

### Mapping of crossovers (COs) and noncrossovers (NCOs) using SNP microarrays.

The whole-genome SNP microarray that detects allele-specific hybridization patterns for about ~13,000 SNP sites distributed across the yeast genome was designed in our previous study (35). Briefly, for each SNP, these Agilent-constructed microarrays contain four oligonucleotides: one pair that hybridizes to the Watson and Crick strands of the W303-1A-derived SNP allele and another that hybridizes to the same strands for the YJM789-derived SNP allele. Tetrads derived from JSC22 were dissected. Genomic DNA from each meiotic product was labeled with Cy5-dUTP, and control DNA from the fully heterozygous JSC24-2 strain (56) was labeled with Cy3-dUTP. The two DNA samples were then hybridized to the SNP microarrays in competition (35). Microarray image acquisition and data extraction were performed as described for the ChIP-chip experiment. Raw data are available at GEO (see below). By measuring the relative ratios of hybridization, we could determine which regions of the spore genome were W303-1A specific and which were YJM789 specific.

### Data analysis.

Identification of transcription factors (TFs) that bind promoter region of ORFs was conducted using the Yeastract website (http://www.yeastract.com/). Mann-Whitney tests and chi-square tests were performed using VassarStat (http://vassarstats.net). *t* tests were performed using the *t* test function in Excel. Fisher exact tests with two-tailed *P* values and Pearson’s correlation analyses were done using GraphPad Prism 6 software. To estimate crossover interference, we calculated the average number of crossovers per tetrad at the various temperatures and used the “sample()” function of R software to place the crossovers randomly on the chromosomes (18). One thousand simulations were done for each temperature.

### Accession number(s).

The raw data determined in this work are available at GEO under accession no. GSE100741 and GSE97667.
