# Supplementary material for: Effects of Temperature on the Meiotic Recombination Landscape of the Yeast Saccharomyces cerevisiae
Source: mBio. 2017 Dec 19;8(6):e02099-17. doi: 10.1128/mBio.02099-17 (PMC5736917; doi:10.1128/mBio.02099-17)
Supplement: TABLE S1 [file mbo006173649st1.docx]

**Table S1.** Comparisons of DSB activities of 22 hottest ORFs at 30^0^ with the same ORFs at other temperatures of sporulation.

| ORFs^a^ | Rank at three temperatures^b^ | | | Up-regulated or down-regulated^c^ | | | 30^0^ HS name; Zhang^d^ | 30^0^ HS name; Miecz.^e^ |
| --- | --- | --- | --- | --- | --- | --- | --- | --- |
|  | 14**^0^** | 30**^0^** | 37**^0^** | 14**^0^**/30**^0^** | 37**^0^**/30**^0^** | 37**^0^**/14**^0^** |  |  |
| *CCT6* | 0.982 | 1 | 0.973 | No | No | No | 88 | 4.28 |
| *NCP1* | 0.814 | 0.89 | 0.875 | Down | No | Up | 178 | 8.03 |
| *SRB2* | 0.874 | 0.889 | 0.871 | No | No | No | 178 | 8.03 |
| *HIS4* | 0.974 | 0.873 | 0.584 | No | Down | Down | 45 | 3.03 |
| *ARE1* | 0.859 | 0.867 | 0.803 | No | No | No | 50 | 3.06 |
| *IMG1* | 0.738 | 0.857 | 0.861 | Down | No | Up | 50 | 3.06 |
| *ERG25* | 0.723 | 0.85 | 0.91 | Down | No | Up | 158 | 7.12 |
| *RUP1* | 0.839 | 0.839 | 0.922 | No | Up | Up | 329 | 15.17 |
| *BUD23* | 0.79 | 0.822 | 0.83 | No | No | No | 50 | 3.06 |
| *DDR2* | 0.792 | 0.821 | 0.614 | No | No | No | 319 | 15.09 |
| *LYS4* | 0.713 | 0.818 | 0.875 | No | No | No | 96 | 4.35 |
| *MHP1* | 0.86 | 0.811 | 0.721 | No | No | No | 219 | 10.10 |
| *HEM12* | 0.758 | 0.81 | 0.89 | No | No | Up | 73 | 4.17 |
| *LSB3* | 0.799 | 0.808 | 0.851 | No | No | No | 125 | 6.06 |
| *CWC25* | 0.753 | 0.807 | 0.832 | No | No | No | 299 | 14.04 |
| *YAT1* | 0.865 | 0.807 | 0.71 | No | No | No | 16 | 1.06 |
| *BNA1* | 0.686 | 0.804 | 0.682 | No | No | No | 223 | 10.13 |
| *YDR186C* | 0.782 | 0.804 | 0.773 | No | No | No | 88 | 4.28 |
| *DIC1* | 0.739 | 0.802 | 0.823 | No | No | No | 269 | NOT HS |
| *CTR1* | 0.741 | 0.795 | 0.861 | No | Up | No | 375 | 16.20 |
| *COA3* | 0.739 | 0.793 | 0.862 | No | Up | Up | 211 | NOT HS |
| *AAC1* | 0.911 | 0.792 | 0.88 | Up | Up | No | 281 | 13.07 |

^a^  These ORFs were the top 22 hot ORFs identified at 30**º**C and were chosen based on the highest ranked hybridization values of oligonucleotides in Dataset S1.

**^b^** Rank values shown in this table is the highest rank if this ORFs have two or more probes in the array.

^c^ “No” means no significant difference in DSBs activities of this ORF between temperatures.

^d^Hotspot name from 30^0^ data of Dataset S3.

^e^Hotspot name from Supp. Table 2 of Mieczkowski *et al.* (2006).
