# Supplementary figures and images for: Effects of Temperature on the Meiotic Recombination Landscape of the Yeast Saccharomyces cerevisiae
Source: mBio. 2017 Dec 19;8(6):e02099-17. doi: 10.1128/mBio.02099-17 (PMC5736917; doi:10.1128/mBio.02099-17)

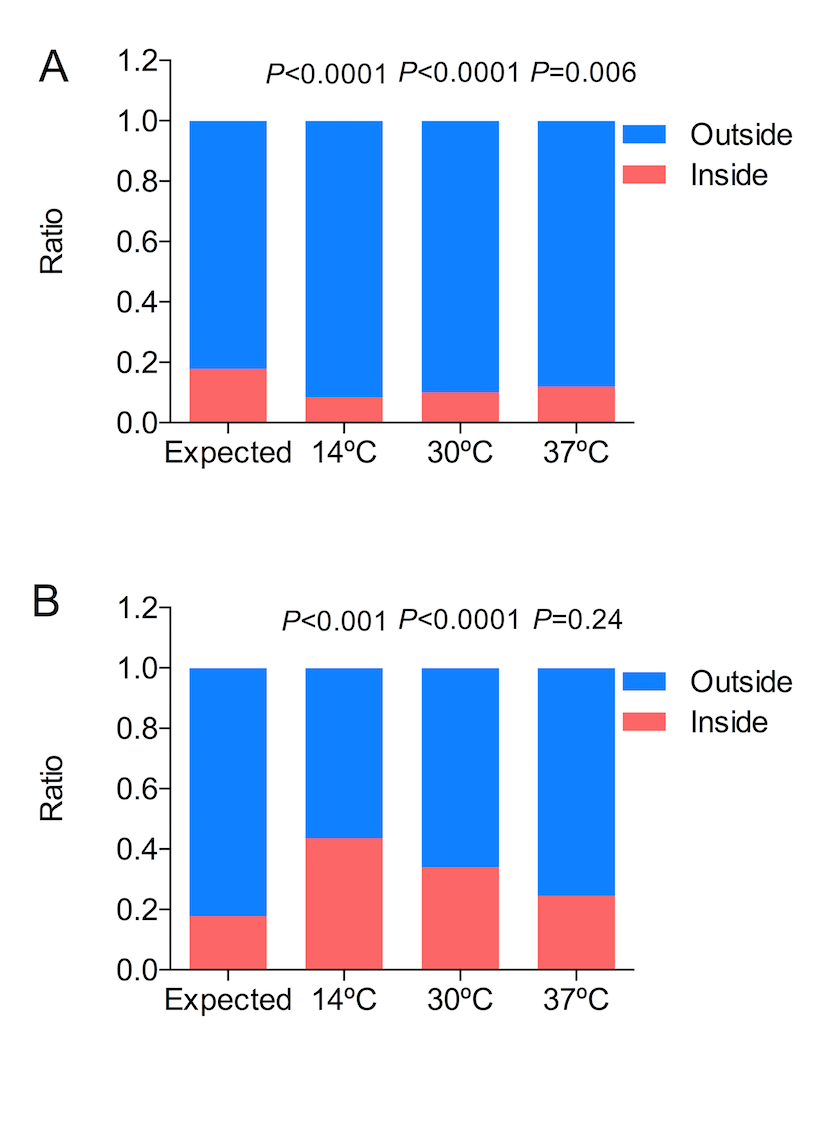

Supplement: FIG S1 [file mbo006173649sf1.tif]

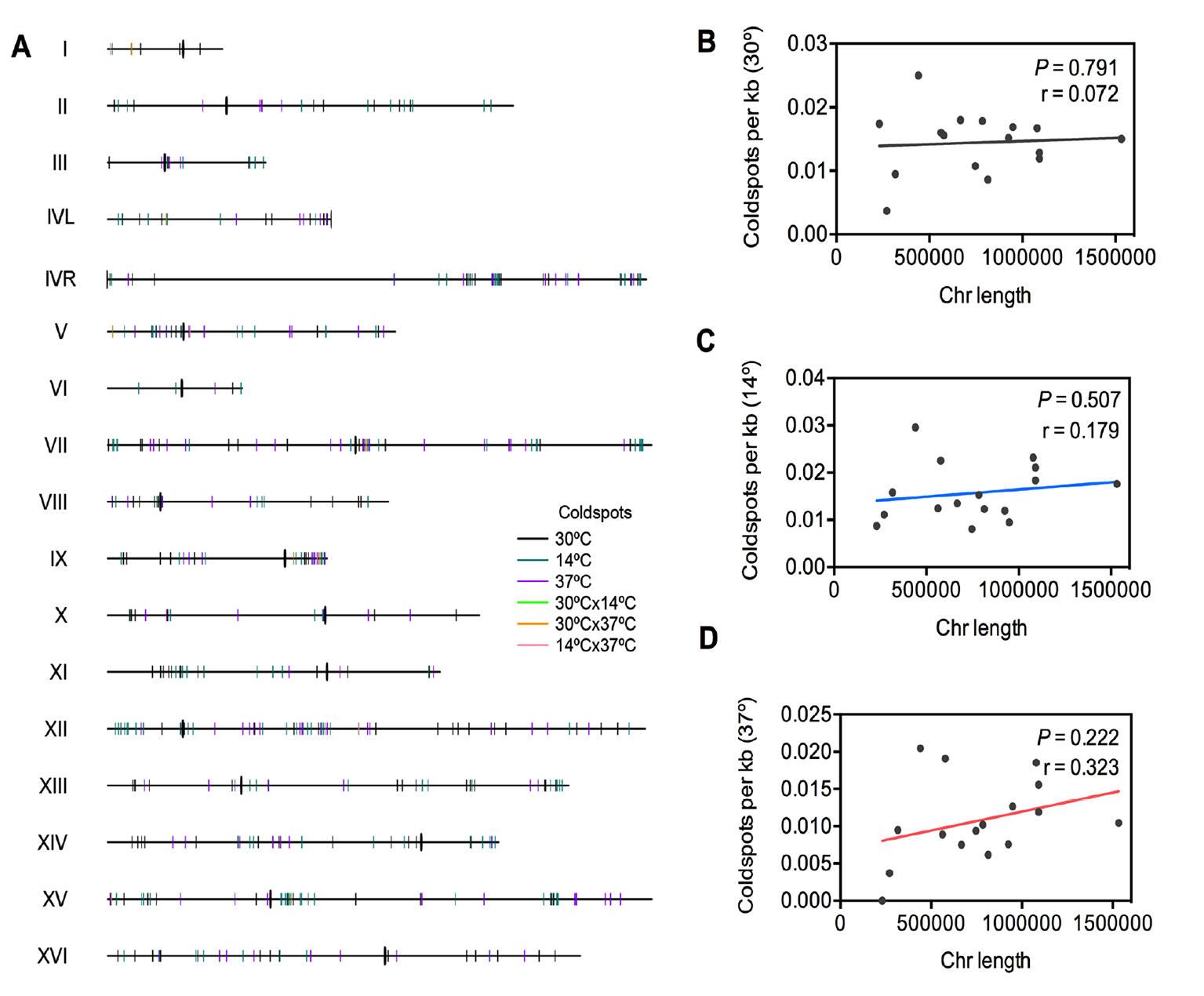

Supplement: FIG S2 [file mbo006173649sf2.tif]

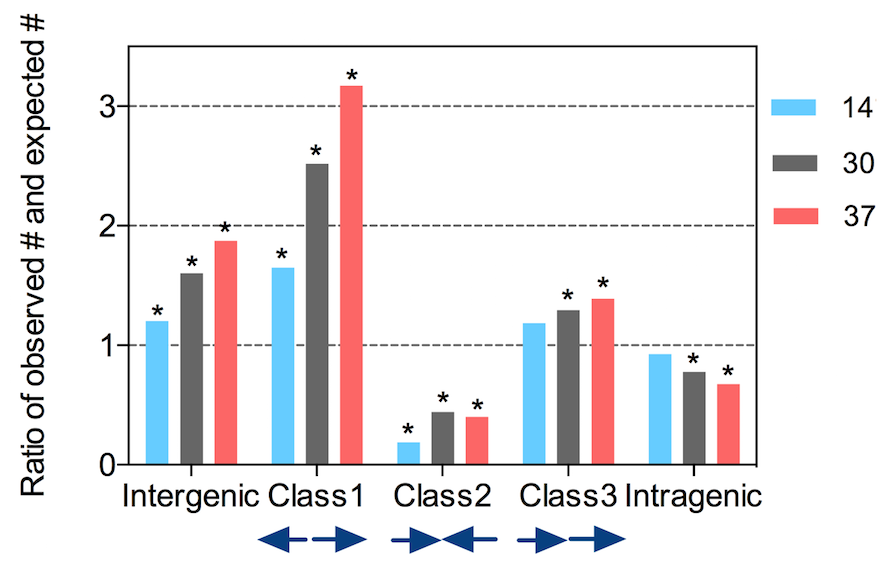

Supplement: FIG S3 [file mbo006173649sf3.tif]

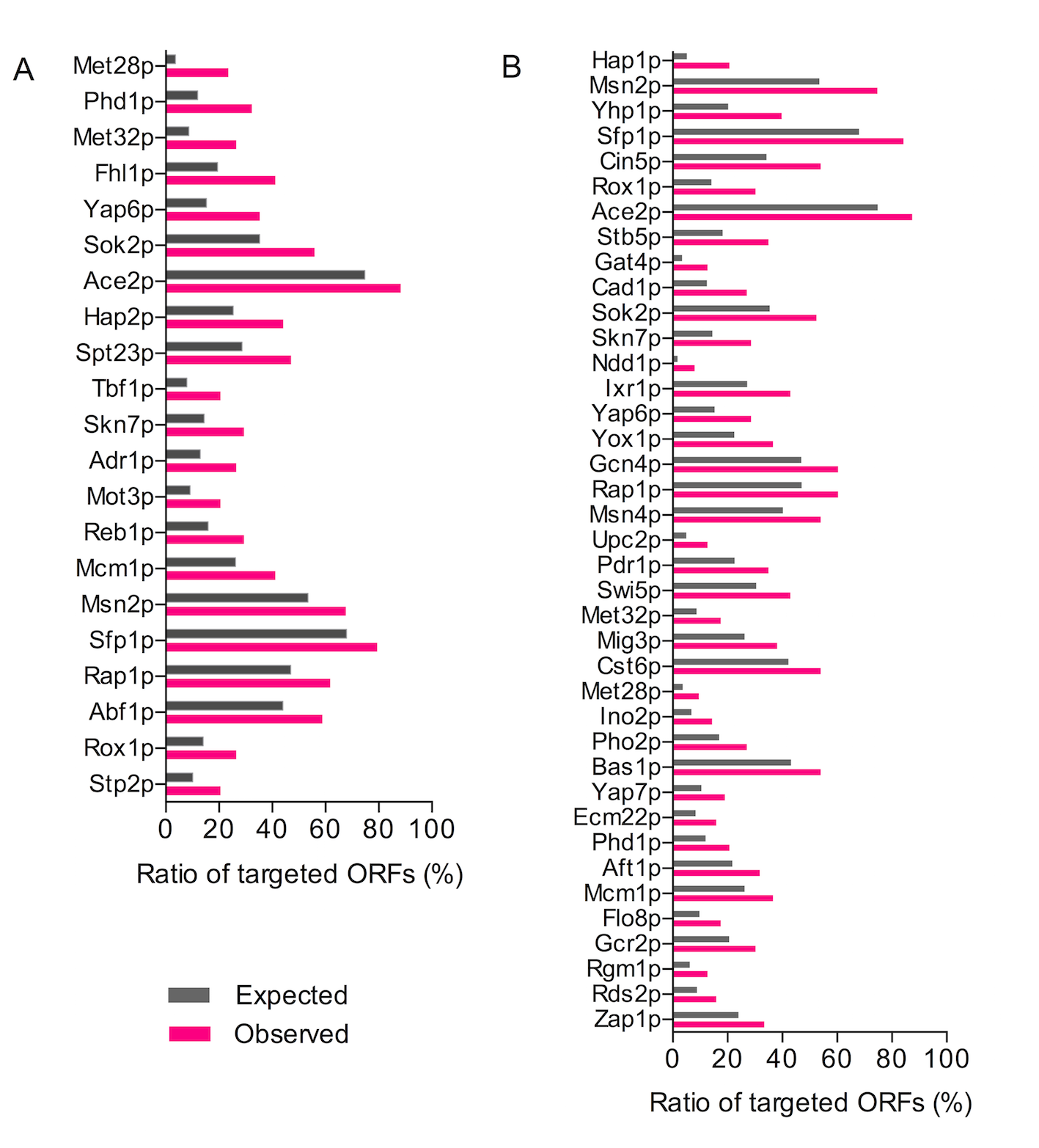

Supplement: FIG S4 [file mbo006173649sf4.tif]
