# Supplementary material for: Effects of Temperature on the Meiotic Recombination Landscape of the Yeast Saccharomyces cerevisiae
Source: mBio. 2017 Dec 19;8(6):e02099-17. doi: 10.1128/mBio.02099-17 (PMC5736917; doi:10.1128/mBio.02099-17)
Supplement: TABLE S2 [file mbo006173649st2.docx]

**Table S2.** Association of hotspots and intergenic/intragenic regions^a^

|  | | Intergenic | Class 1 | Class 2 | Class 3 | Intragenic |
| --- | --- | --- | --- | --- | --- | --- |
| Probes # | | 4021 | 1423 | 593 | 2005 | 10816 |
| 14**^0^** | Observed inside # | 173 | 84 | 4 | 85 | 358 |
|  | Observed outside # | 3848 | 1339 | 589 | 1920 | 10458 |
|  | Expected inside # | 144 | 51 | 21 | 72 | 387 |
|  | Expected outside # | 3877 | 1372 | 572 | 1933 | 10429 |
|  | p-value | 1.4E-02 | 2.4E-06 | 1.4E-04 | 1.1E-01 | 1.3E-01 |
| 30**^0^** | Observed inside # | 221 | 123 | 9 | 89 | 288 |
|  | Observed outside # | 3800 | 1300 | 584 | 1916 | 10528 |
|  | Expected inside # | 138 | 49 | 20 | 69 | 371 |
|  | Expected outside # | 3883 | 1374 | 573 | 1936 | 10445 |
|  | p-value | 6.2E-13 | 3.3E-27 | 1.0E-02 | 1.3E-02 | 1.1E-05 |
| 37**^0^** | Observed inside # | 222 | 133 | 7 | 82 | 215 |
|  | Observed outside # | 3799 | 1290 | 586 | 1923 | 10601 |
|  | Expected inside # | 118 | 42 | 17 | 59 | 319 |
|  | Expected outside # | 3903 | 1381 | 576 | 1946 | 10497 |
|  | p-value | 4.5E-22 | 2.8E-46 | 1.1E-02 | 2.4E-03 | 3.9E-09 |

^a^The number of elements (oligonucleotides) on the microarray within the various categories of intergenic and intragenic regions were used to generate the expected numbers. The expected numbers were produced by multiplying the number of “hot” elements by the fraction of the elements within the various classes. For example, the number of elements expected in the Intergenic class of the 14^0^ data was 531 x (4021/14837)=144. Class 1, 2, and 3 intergenic regions are between divergently-transcribed genes, convergently-transcribed genes, and genes transcribed in the same direction, respectively. p values were determined by chi-square tests.
